# Flexible and Electrically Tunable Plasmons in Graphene–Mica Heterostructures

**DOI:** 10.1002/advs.201800175

**Published:** 2018-06-16

**Authors:** Hai Hu, Xiangdong Guo, Debo Hu, Zhipei Sun, Xiaoxia Yang, Qing Dai

**Affiliations:** ^1^ Division of Nanophotonics CAS Center for Excellence in Nanoscience National Center for Nanoscience and Technology Beijing 100190 P. R. China; ^2^ University of Chinese Academy of Sciences Beijing 100049 P. R. China; ^3^ Department of Electronics and Nanoengineering Aalto University FI‐00076 Aalto Finland; ^4^ QTF Centre of Excellence Department of Applied Physics Aalto University FI‐00076 Aalto Finland

**Keywords:** flexible plasmons, graphene–mica heterostructures, graphene plasmons, mid‐infrared plasmons

## Abstract

Flexible plasmonic devices with electrical tunability are of great interest for diverse applications, such as flexible metamaterials, waveguide transformation optics, and wearable sensors. However, the traditional flexible metal–polymer plasmonic structures suffer from a lack of electrical tunability. Here the first flexible, electrically tunable, and strain‐independent plasmons based on graphene–mica heterostructures are experimentally demonstrated. The resonance frequency, strength, quality factor, electrical tunability, and lifetime of graphene plasmons exhibit no visible change at bending radius down to 1 mm and after 1000 bending cycles at a radius of 3 mm. The plasmon‐enhanced infrared spectroscopy detection of chemicals is also demonstrated to be unaffected in the flexible graphene–mica heterostructures. The results provide the basis for the design of flexible active nanophotonic devices such as plasmonic waveguides, resonators, sensors, and modulators.

## 1 Introduction

Graphene plasmon can manipulate electromagnetic signals at deep‐subwavelength scale with ultrahigh field confinement, driven by its quasiparticle Dirac fermions that obey a linear dispersion.^1^, ^2^, ^3^, ^4^ Due to its variable Fermi level, graphene plasmon resonance can be electriallly tuned in the range from the terahertz to the infrared.^5^, ^6^, ^7^, ^8^, ^9^ These extraordinary properties make graphene plasmon a promising platform for strong light‐matter interactions,^10^, ^11^, ^12^, ^13^ deep‐subwavelength metamaterials,^14^, ^15^, ^16^ and active nanophotonic devices such as surface enhanced infrared absorption (SEIRA) applications,^17^, ^18^, ^19^, ^20^, ^21^ tunable notch filters,^22^, ^23^ modulators,^24^, ^25^ and waveguides.^26^, ^27^, ^28^, ^29^

There is widespread theoretical interest in flexible graphene plasmonic devices such as bendable waveguides^30^, ^31^, ^32^, ^33^ and wave splitters^31^ due to the robust and ultraflexible mechanical property^34^ of single‐atomic‐thick graphene in contrast to its traditional 3D metallic plasmonic structure counterpart. However, experimental realization of flexible graphene plasmonic devices has remained elusive for two main reasons. First, proper flexible substrate is absent. The conventional flexible substrates typically have strong absorption (nearly saturated absorption) in the mid‐infrared range, which would significantly impede their applications (such as SEIRA and photodetector); second, it is difficult to precisely fabricate periodical graphene nanostructures (about 100 nm) on flexible and mechanically compliant substrates via nanofabrication techniques such as electron beam lithography (EBL) or focused ion beam etching.^35^ The lack of dielectric properties of the traditional flexible substrates (such as organic films) also restricts their usage in the electrical tunable graphene plasmons.

Here, we experimentally demonstrate flexible and electrically tunable plasmonic devices with graphene–mica heterostructures. The mica thin sheet acts as a highly flexible and transparent substrate with atomically flat surface for graphene.^36^, ^37^ The plasmon responses (such as resonance frequency, extinction intensity, quality factor, and electrical tunability) of our mica heterostructures remain nearly unchanged even with a bending radius of 1 mm or with relative fatigue strength (>1000 bending cycles). Electromagnetic simulation results reveal that graphene plasmon can bear strong curvature even down to 150 nm due to its ultrastrong field confinement. Based on these robust properties, our flexible graphene plasmonic devices enable applications in SEIRA, which are independent of bending.

## 2 Results and Discussion

**Figure 1:**
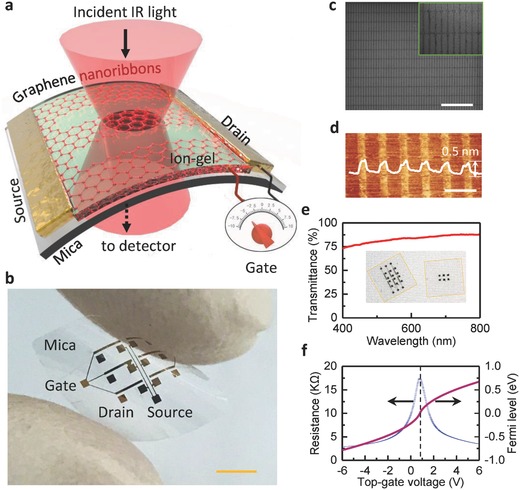
Flexible graphene–mica heterostructure plasmonic device. a) Schematic illumination of plasmon excitation and detection of our flexible graphene–mica plasmonic device. b) Photograph of the graphene–mica heterostructure plasmonic devices. The scale bar is 2.5 mm. c) SEM image of the GNRs. The scale bar is 2 µm. Inset: zoom‐in view of nanoribbons. d) AFM image of GNRs. Inset: line‐scan profile of the image. The GNRs have a uniform thickness of about 0.5 nm on the mica substrate. The scale bar is 0.2 µm. e) The transmittance of our flexible graphene–mica heterostructure plasmonic devices in the visible range. f) Typical transfer characteristics and Fermi level of graphene controlled by the ion–gel top gate.

The working principle of our flexible graphene plasmonic device is illustrated in **Figure** **1**a. The graphene nanoribbon (GNR) plasmonic devices were fabricated using Si substrate‐back support method to overcome the problems of the weak support and mechanically compliant of flexible mica sheet (Figure S1, Supporting Information). Figure 1b shows an optical photograph of graphene plasmonic devices with six pairs of electrodes. Figure 1c,d shows scanning electron microscope (SEM) and atomic force microscope (AFM) images of the GNRs. Surface morphology of the GNRs clearly indicates that our method enables accurately, uniformly, and damage‐freely fabrication of GNRs in the heterostructures with ribbon width below 100 nm. Another significant advantage of the mica substrate is its optical transparency. The optical transmittance of our flexible devices is higher than 75% in the whole visible range (Figure 1e). Figure 1f shows that ion–gel top gate can shift Fermi level of graphene in a wide range (>0.8 eV) with a small gate voltage change (<8 V) due to the high capacitance of Debye layer formed at the ion–gel/graphene interface (Figure S2, Supporting Information).^38^ The effective control of carrier density of graphene can lead to the broad tunability of graphene plasmon.^7^

The plasmonic properties of graphene were characterized by Fourier transform infrared microscopy (FTIR). In GNRs, localized plasmon can be directly excited due to the wave‐vector match between external free photons and surface plasmon polaritons via ribbon edge reflection.^39^ Based on the electrical tunability, an in situ observation method was used to obtain the extinction spectra of plasmon, *T* = 1 − *T_E_*_F_/*T*_CNP_, where *T*_CNP_ and *T_E_*_F_ are the transmission spectra detected at the charge neutral point (CNP) and *E*_F_ of graphene, respectively.

**Figure 2:**
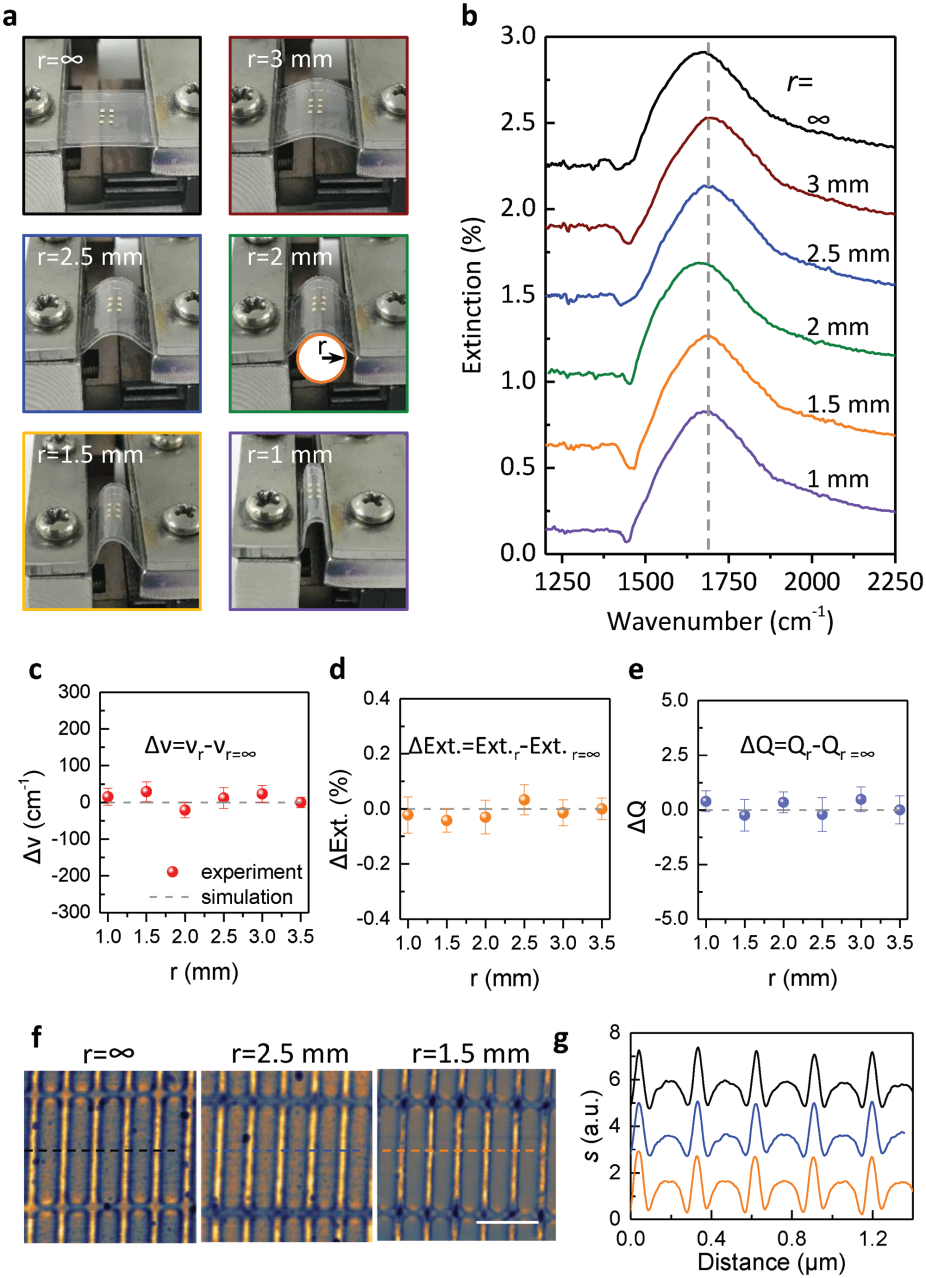
Bending‐independent performance of our flexible graphene plasmonic devices. a) Photographs of our devices with different bending curvature radii (*r*) ranging from *∞* (flat) to 1 mm. b) Experimental extinction spectra of our graphene–mica plasmonic devices with different bending radii corresponding to (a). c–e) The variation of resonance frequency (ν), extinction intensity (Ext.) and quality factor (*Q*) of our flexible graphene–mica plasmonic devices as a function of bending radius, respectively. The red dots and gray dashed lines represent experimental and simulation results, respectively. f) Nanoinfrared imaging of GNRs in our devices with different bending radii including *r* = *∞*, 2.5 and 1.5 mm, respectively. The scale bar is 0.5 µm. g) Line profiles across the plasmonic fringes, corresponding to the dashed lines in (f).

We performed a series of flexibility tests of our devices. **Figure** **2**a displays the graphene–mica (≈120 µm in thickness) device could be curved with different bending radii from 3.5 to 1 mm by a home‐made bending system. Plasmonic spectra responses of the flexible device at various bending radii were measured, as presented in Figure 2b. The dominant features of these extinction responses at different bending radii are almost identical. There is a prominent peak in each extinction spectrum, which origins from the graphene plasmon resonance. The resonance frequency is indicated by a vertical line. Notably, even when the bending radius was down to 1 mm, we did not observe any damage of the device and plasmon could be effectively excited and does not show a significant change in the spectrum. The results fully confirm that our plasmonic device is flexible and can withstand strong bending while keeping its plasmonic properties. This further demonstrates the uniaxial strain of graphene is fairly minor in our bending experiments (<0.1%),^40^ thus the strain‐induced bandgap engineering effects of graphene can be ignored.^41^

Resonance frequency (ν), extinction intensity (Ext.), and quality factor (*Q*) are the three most important indicators in practical applications because they are strongly associated with the enhancement and confinement of local electric field which are at the heart of sensors and other nano‐optical devices.^42^, ^43^, ^44^ These characters of our flexible devices at various bending radii were extracted from the experimental results and compared with simulation results (Figure S3, Supporting Information). The variation of resonance frequency for different bending radii is ±25 cm^−1^. These values are very small compared to the resonance frequency (≈1600 cm^−1^) and the full width at half maximum (FWHM, ≈330 cm^−1^) of the plasmon resonance peak (Figure 2c). We also observe that the change of extinction intensity (±0.05, Figure 2d) and quality factor (±0.5, Figure 2e) as a function of bending radius is also negligible. These results further confirm that our device is flexible and its plasmonic performance at different bending radii is almost identical (i.e., bending independent).

In order to directly observe the localized plasmons on the GNRs with various bending radii, we performed scattering near‐field scanning optical microscopy (s‐SNOM) imaging of the device.^26^, ^27^ The excitation frequency is 895 cm^−1^, which could stimulate plasmon on graphene effectively. Figure 2f shows representative near‐field images from 2D scan of the top position of the graphene device with varied bending radii of *r* = *∞*, 2.5 and 1.5 mm. The bright fringes aligned parallel to the ribbon edges are formed from constructive interference by tip launched plasmon and edge‐reflected plasmon.^45^ Figure 2g plots line profiles across the fringes, extracted from the s‐SNOM images. At different bending radii, the intensity and distribution of collected near‐field signals of localized plasmon resonance are identical to that of the flat state. These near‐field results also clearly validate the bending‐independent performance of our flexible graphene–mica plasmonic devices. This is in agreement with the far‐field results (Figure 2b) and the numerical calculations (discussed in the next paragraph).

**Figure 3:**
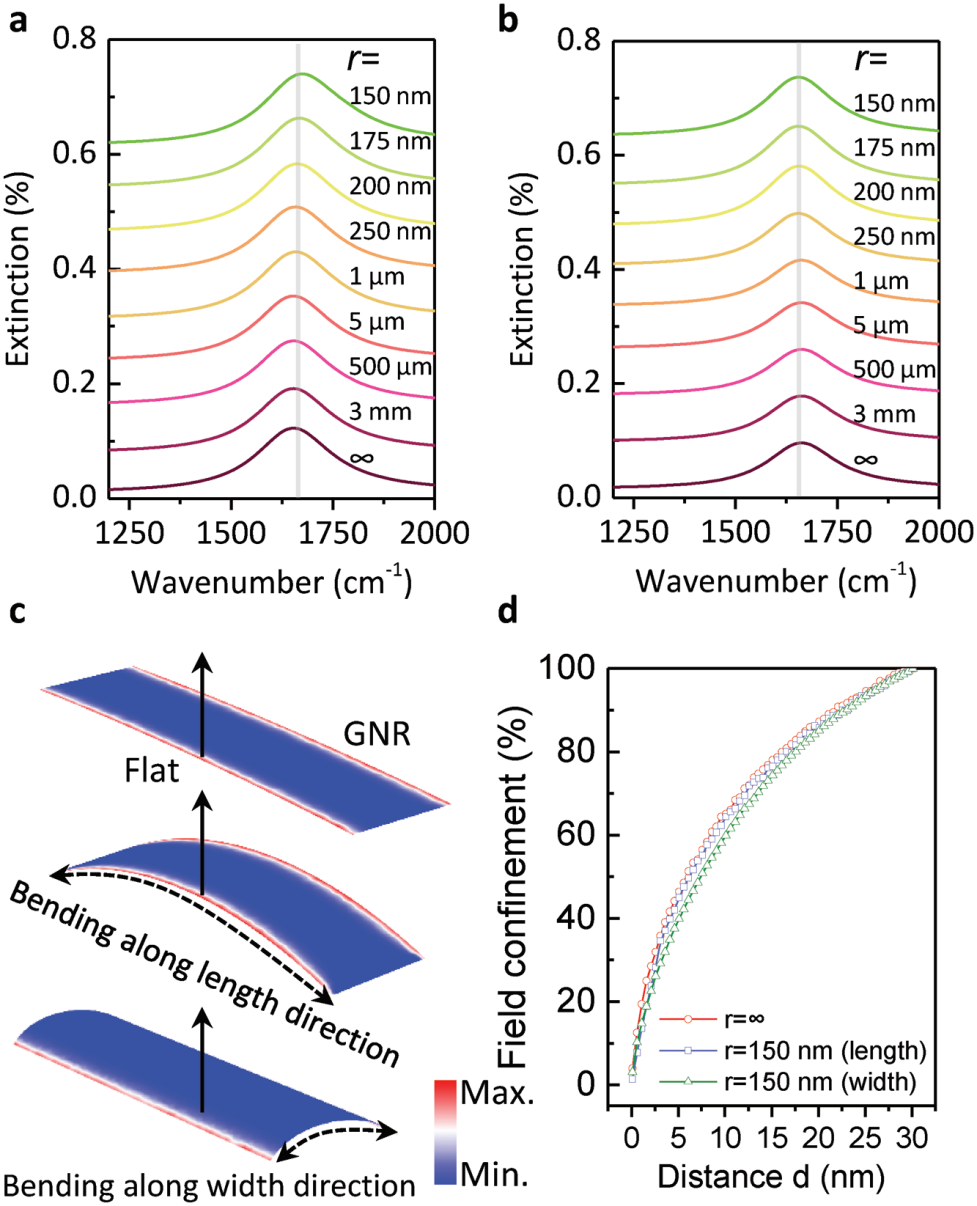
Simulation results of flexible graphene plasmon. a,b) Calculated extinction spectra of free‐standing graphene plasmon on arc nanoribbons bending along the length and width directions, respectively. The corresponding bending radii are indicated. c) Electric charge distribution of the plasmonic modes, corresponding to the resonance modes in (a,b). d)The field confinement calculated from (c) along direction indicated by the arrows.

To understand the experimental results, we conduct simulation (**Figure** **3** and Figure S3, Supporting Information) under various bending radii by using a frequency‐domain finite‐element model (for more details, refer to the Experimental Section).^46^ For simplicity, we focus on curved free‐standing GNRs, and bendings along both directions (i.e., the length and width of directions) are considered. Kubo formula is used to calculate the surface conductivity of graphene (Equation 1 in the Experimental Section).

The calculated extinction spectra of the GNRs with different bending radii along the length and width direction are plotted in Figure 3a,b and Figure S3a,b in the Supporting Information, respectively. The extinction spectra keep the same even when the nanoribbon bends with a bending radius of 150 nm along both length and width directions. The calculated variation of resonance frequency, extinction intensity, and quality factor of the graphene plasmon are extracted and plotted in Figure 2c–e, agreeing well with experimental results. We extract the near‐field intensity confinement as a function of the distance *d* from out‐plane of graphene (Figure 3d and Figure S3e, Supporting Information). In all three conditions, about 80% of the plasmon energy is confined within a volume extending a distance 20 nm outside nanoribbon, which is almost irrelevant to bending deformation of GNRs. The ultrastrong field confinement keeps the spatial overlap of plasmon in curved graphene low, which results the maintained performance of the plasmonic devices with radius above 150 nm, as discussed above. We also calculate that the limit radius of curvature for flexible graphene plasmon with stability can be 3 nm (for details, see Section S3 in the Supporting Information). The ultrastrong field confinement also protects the plasmonic resonance energy from curvature‐induced radiative energy loss which could affect the charge‐density oscillation efficiencies.^30^

**Figure 4:**
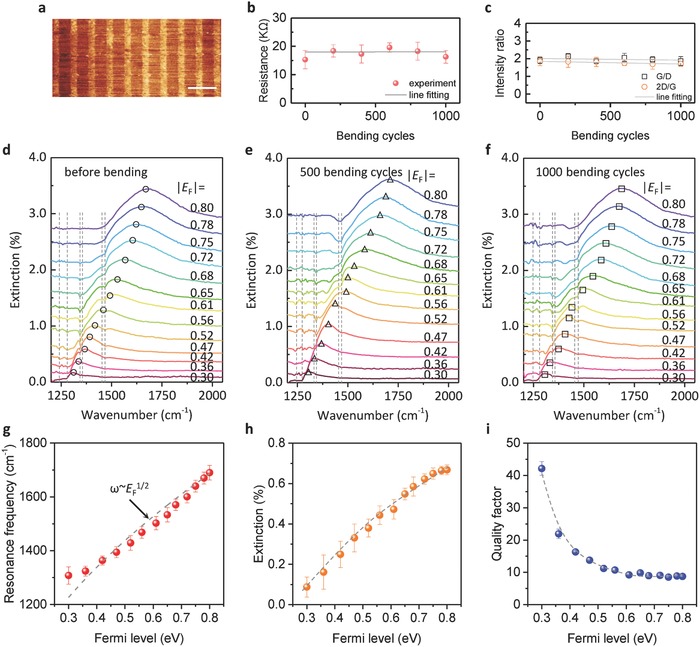
Broad tunability and high durability of our flexible graphene–mica plasmonic device. a) AFM image of the GNRs after 1000 bending cycles. The scale bar is 0.2 µm. b) Graphene resistance as a function of different bending cycles with a bend radius of 3 mm. Horizontal straight line: a linear fit. c) *G*/*D* and 2*D*/*G* ratio value of Raman spectrum of GNRs as a function of different bending cycles. Straight lines: linear fit. d–f) Extinction spectra of graphene plasmon device at various values of *E*_F_ controlled by ion–gel top gate before bending testing, after 500 and 1000 bending cycles, respectively. The plasmon resonance peaks are indicated by circles, triangles, and squares. The vertical dashed lines indicate the molecular vibrational modes of the ion gel. g–i) The plasmon resonance frequency, extinction intensity and quality factor plotted as a function of the Fermi level of the graphene–mica plasmonic device after 1000 bending cycles. The gray dotted curves are guides to the eye.

We further studied the flexibility of our graphene–mica heterostructure plasmonic devices as a function of the bending cycle at 3 mm curvature radius using a linear motor. After repeated bending of 1000 cycles in 75 min, the morphology of GNRs was in good shape without measurable change (**Figure** **4**a and Figure S4, Supporting Information). The electrical performance of the device showed negligible variation and the resistance maintains at ≈15 KΩ even after 1000 bending cycles (Figure 4b and Figure S5a, Supporting Information). Raman spectra showed that the ratio of relative intensity of *G*/*D* and 2*D*/*G* changes slightly (<12%) (Figure 4c and Figure S5b, Supporting Information). We attribute the good flexibility performance of our device is due to the high flexibility of both mica and graphene. Mica possesses high flexibility because it is a layered framework of aluminosilicates,^47^ and the excellent mechanical properties of graphene inherits from the single atomic layer of carbon with low bending stiffness, ultrahigh Young's modulus (≈1 TPa) and intrinsic strength (≈130 GPa).^48^

Plasmonic properties were investigated during and after different bending cycles. Even after experiencing 1000 bending cycles, the device was able to effectively excite plasmon and show unaffected electrical tunability. Figure 4d–f shows the extinction spectra of the flexible graphene–mica plasmonic device at different *E*_F_ before, and after 500 and 1000 bending cycles, respectively. For all the three conditions, one prominent plasmonic resonance peak appears for each spectrum, and can be dynamically tuned by the ion–gel top gate, which shifts toward blue with |*E*_F_| increasing. The resonance frequency varies from 1303 to 1680 cm^−1^ when *E*_F_ varies from 0.3 to 0.8 eV (corresponding effective gate voltage (*V*_g_ − *V*_CNP_) varies from 0.5 to 6.5 V), with increasing plasmon intensity. The shallow dips on the spectra, located at the molecular vibrational modes of ion–gel, stem from their interaction to the graphene plasmons.^49^

The effects of bending on the electrical tunability of the graphene plasmon are concluded in Figure 4g–i. The resonance frequencies at the same Fermi level before and after bending cycles keep unchanged, and their deviation from the mean values are less than 26 cm^−1^ which is only 6.4% of the tunable bandwidth (from 1275 to 1680 cm^−1^). The extinction intensity of plasmon is proportional to Fermi level due to more carriers involved in the resonance oscillation, as shown in Figure 4h. The intensity can be regulated over one order of magnitude from 0.07% to 7.2% under the bending states and the mean standard error is only 0.45%. Figure 4i primarily suggests that the highest quality factor is up to 45 and the mean variation is only 2.2% after bending process. In additional, mica film also can be used as effective dielectric layer to electrically tune graphene plasmon based on common back gate setup due to high‐K dielectric of mica (ε is about 5–8).^50^

**Figure 5:**
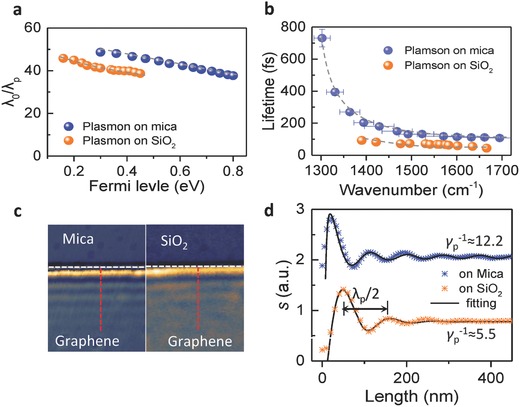
Highly confined and long lifetime of flexible plasmon in our graphene–mica heterostructure. a,b) Graphene plasmon confinement (λ_0_ */λ*_p_) and lifetime (*t*) as a function of Fermi level, respectively. Results from SiO_2_ substrate is also plotted for comparison (Figure S6, Supporting Information). The gray dashed curves are guides to the eye. Error bar is calculated from the results in Figure 4d–f. c) Near‐field amplitude s (ω = 895 cm^−1^) images of graphene plasmon on mica and SiO_2_ substrates. The scale bar is 0.2 µm. d) Line profiles of plasmon intensity *s* across the graphene edge (the white dash lines in c).

Graphene plasmon has been demonstrated to possess ultrahigh Field localization (wave “shrinkage”) which is defined as how small the supported plasmon wavelengths are compared to the free space wavelength (λ_0_/λ_p_). It is crucial in a wide range of applications from biochemical sensors to deep‐subwavelength metamaterials^51^, ^52^ Plasmons with multiple reflections between the two edges of GNRs are similar to Fabry‐Perot resonances. Then the graphene plasmon wavelengths (λ_p_) are extracted following refs. 39, 53. **Figure** **5**a displays the field localization of plasmon in our flexible graphene–mica heterostructure device. λ_0_/λ_p_ is as high as 48 with resonant energy at 1300 cm^−1^ (|*E*_F_| = 0.3 eV), which is slightly higher than that of plasmon on SiO_2_ (i.e., 41) at same Fermi level and with same ribbon width (Figure S6, Supporting Information). The ratio of λ_0_/λ_p_ increases as the Fermi level decreases following the graphene plasmon dispersion relation of *E*∝*E*_F_^1/2^. The plasmon lifetime can be calculated via *t* = 2*ħ*/*Γ*, where *Γ* is the FWHM of the resonance peaks extracted from the far field extinction spectra using Fano curve fitting (Figure S7a, Supporting Information).^52^ Figure 5b comparatively shows the plasmon lifetime of graphene supported on mica and on SiO_2_ at different Fermi levels with the same ribbon width (Figure S6, Supporting Information). The plasmonic lifetime decreases as the resonance energy increases, which relates to the plasmon–phonon coupling.^52^, ^54^ There is strong infrared‐active phonon absorption from ≈950 to ≈1200 cm^−1^ (1168 cm^−1^) in our mica (SiO_2_) substrate, which resulted strong plasmon–phonon coupling in our graphene–mica (graphene/SiO_2_) devices (Figure S8, Supporting Information). The plasmon–phonon coupled modes have longer lifetime as they approach the phonon energy since they gain more properties from the phonons.^52^, ^54^ The longest lifetime of graphene plasmon on SiO_2_ substrate was about 100 fs, however, the lifetime of our graphene–mica heterostructure approaches 800 fs at around 1300 cm^−1^. This is mainly due to the long lifetime of phonons in mica for its 2D crystalline structure which results less damping paths than the SiO_2_ substrate. Another feature is that the plasmon lifetime of our graphene–mica device is still larger than that of the graphene/SiO_2_ in the higher frequency range (>1580 cm^−1^) where the effects of substrate phonon fades. This lower damping may contribute to the atomic flat surface of mica, which introduces less electron–electron scattering.^55^

We also performed the real‐space imaging of plasmon fields of the flexible graphene–mica devices, which further demonstrated the far‐field spectral results. Mechanically exfoliated graphene is used to obtain better near‐field signal (Figure S9, Supporting Information). Figure 5c shows representative near‐field images (third‐order demodulated harmonics of the near‐field amplitude) on mica and SiO_2_ substrates, respectively. The fringes parallel to the graphene edge are formed by the interference of tip‐launched forward propagating plasmons and the partially reflected plasmon waves by the edge, and the oscillation period equals to λ_p_/2. Figure 5d plots line profiles of the near‐field signals across the edge at the excitation wavelength of 895 cm^−1^ (λ_0_ = 11.12 µm). The λ_p_ in our graphene–mica heterostructure is 196 nm, corresponding to a wavelength confinement of ≈57, while the λ_p_ of the graphene‐SiO_2_ device is 220 nm and the confinement of ≈51. This is consistent with our far‐filed experimental observations. The inverse damping ratio γ_p_^−1^ = Re (*q*_p_)/Im (*q*_p_) is also calculated by fitting with the method described by Woessner et al.^51^ The γ_p_^−1^ of graphene on mica is 12.2 which is approximately two times larger than that on SiO_2_, which is in accord with the lifetime results from the far‐field calculation. This is because graphene on the atomically flat mica surface has reduced electron scattering loss and thus small plasmon damping, similar to the case of the graphene‐BN heterostructure. It is reasonable to deduce that graphene encapsulated between two mica films could realize lower plasmon damping combined with stronger field confinement.^51^

**Figure 6:**
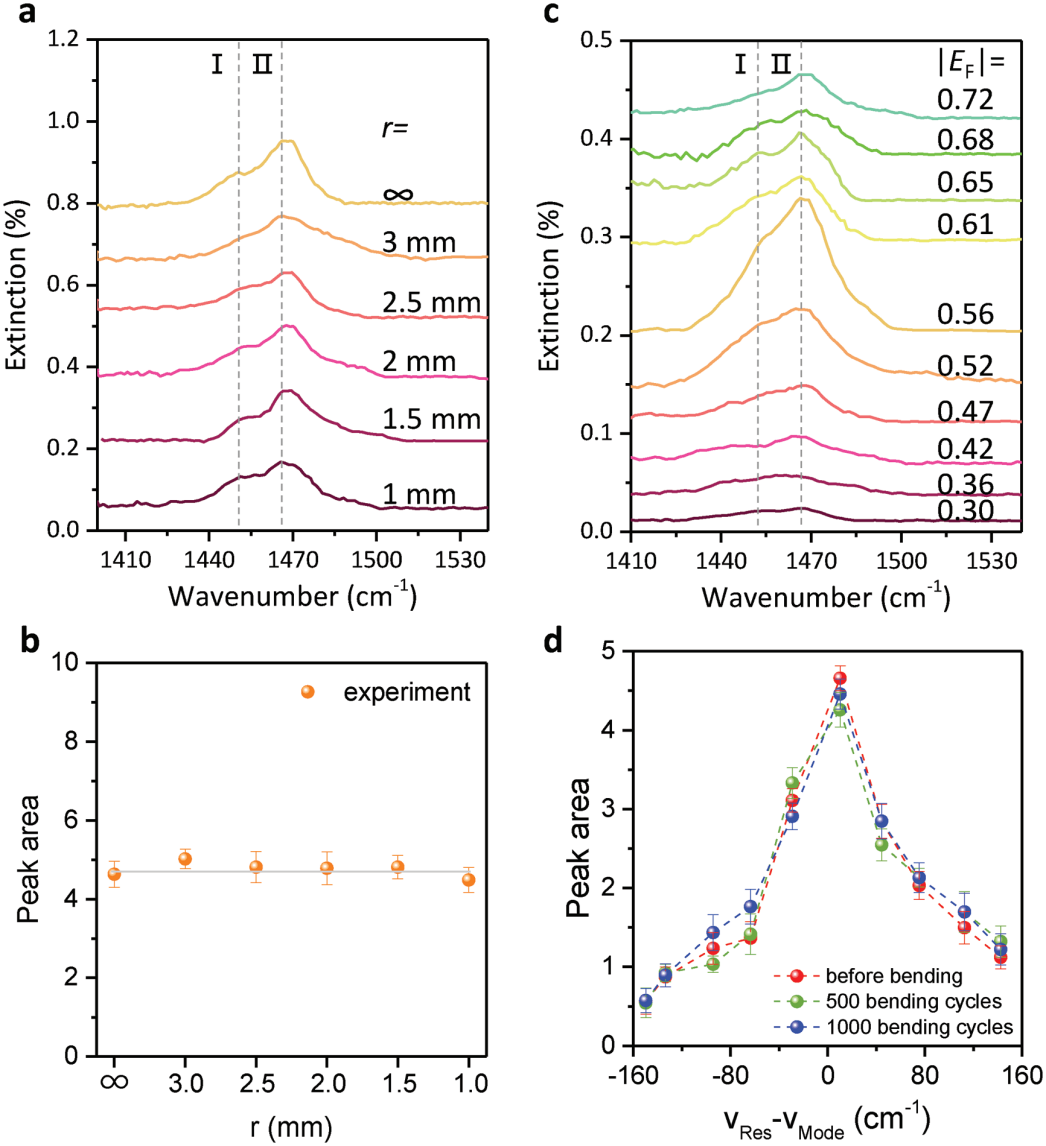
The unchanged SEIRA function of our flexible plasmonic device. a) The plasmon‐induced vibrational mode response of two typical vibrational modes (δ(CH_2_)) of the ion gel as a function of different bending radius. I: δ(CH_2_)_S_ + δ(CH_2_)_a_, II: δ(CH_2_)_S_ − δ(CH_2_)_a_). δ indicates bending modes and the suffixes a and s represent the symmetric and anti‐symmetric modes, respectively. b) The enhanced peak area of the modes I and II calculated from (a) as a function of different bending radii. The error bars in the plots are standard deviation from large numbers of measurements. c) The plasmon‐induced response of the modes I and II in the extinction spectra of graphene plasmon with 3 mm bending radius at different Fermi levels after 1000 bending cycles. d) The enhanced peak area of the modes I and II with 3 mm bending radius as a function of the difference between the mode (*v*_Mode_) and plasmon resonance peak (*v*_Res_) before bending testing (red), after bending process with 500 cycles (green) and 1000 cycles (blue), respectively.

The unaffected graphene plasmons in our flexible graphene–mica heterostructure devices guarantee the sensitive SEIRA applications. Here, the ion–gel film which has characteristic infrared absorption peaks acts as the analyte for the SEIRA applications.^49^ As shown in Figure 4d–f, when the graphene plasmon resonances come across the molecular infrared vibrational modes (dashed vertical lines), they interact with each other destructively and yield dips in the plasmon resonance peak. The signals of the molecules within the plasmonic hotspots are enhanced by the strong plasmonic resonance. This coupling is the basic mechanism of the SEIRA. Here, we selected modes I: δ(CH_2_)_S_ + δ(CH_2_)_a_ and II: δ(CH_2_)_S_ − δ(CH_2_)_a_) as the probe to illustrate the SEIRA function of the flexible devices. The plasmon‐induced molecular vibrational signals are extracted from the extinction spectra with graphene Fermi level of 0.65 eV following the method in Ref. (**Figure** **6**a). When the bending radius changes, we can see that the plasmon‐induced signals remain since the plasmonic properties do not change, as exhibited in Figure 2 and Figure S10 in the Supporting Information. The peak area values (Figure S7b, Supporting Information) of the modes plotted as a function of different bending radius in Figure 6b further demonstrate that the signal enhancement is not affected.

The plasmon‐induced molecular vibrational signals of the modes I and II are also extracted from the extinction spectra with 3 mm bend radius at different Fermi levels before bending testing, after 500‐cycle and 1000‐cycle bending. The set of spectra after 1000 bending cycles are representatively displayed in Figure 6c. When the absolute value of the Fermi level reaches 0.56 eV, the plasmon resonance peak (at ≈1490 cm^−1^) approaches the modes I and II, and the enhancement largely increases. As the |*E*_F_| increases or decreases, the plasmon resonance peak moves away from the modes I and II, and the enhanced vibrational signals decrease. The peak areas of these enhanced vibrational signals are plotted in Figure 6d as a function of the frequency difference between the vibrational mode and graphene plasmon. For all the three kinds of conditions, before bending testing (red), after bending process with 500 cycles (green) and 1000 cycles (blue), the enhanced peak areas increase as the |*v*_Mode_ − *v*_Res_| values decrease . These results strongly demonstrate the stability and duration of our flexible graphene–mica heterostructure based plasmonic devices for the SEIRA applications.

## 3 Conclusions

In conclusion, flexible and electrically tunable plasmonic devices were demonstrated with our graphene–mica heterostructure for the first time. The graphene–mica devices exhibit high flexibility (with bending radius down to 1 mm). High mechanical durability and electrical tunability have also been demonstrated due to the excellent mechanical properties of graphene and mica. Electromagnetic simulation reveals that the bending radius can be down to about 150 nm with negligible effects on the graphene plasmon response since the ultrahigh confinement of graphene introduces weak coupling or damping in the bending structures. Using infrared vibrational modes of ion–gel as probe analyte, the SEIRA function of the flexible devices at different bending conditions are demonstrated, which is unaffected by bending. Our graphene–mica heterostructure strategy provides an excellent platform for photonic devices such as wearable sensors,^56^ and enhanced photodetectors.^57^

## 4 Experimental Section

*Fabrication of Flexible Graphene–Mica Plasmonic Device*: Graphene was grown on copper foil via chemical vapor deposition and was then transferred on to mica substrate by a common wet transfer technique. Next, ethyl lactate and about 350 nm thick PMMA solution was spin coated on to a Si substrate and the prepared graphene–mica sheet was fastened onto this Si substrate before the solvent of PMMA was dried because it was supplied as a bonding layer. The sample was baked at 40 °C for 30 min until it was completely dry. Another 80 nm PMMA layer was spin coated on graphene–mica to be a mask and a 30 nm Al film was deposited on it as a conductive layer. GNR arrays were patterned via 100 keV EBL (Vistec 5000+ES, Germany). The sample was soaked in the alkali solution to remove the surface aluminum layer. The exposed PMMA was developed in one‐to‐three methyl isobutyl ketone (MIBK)/ isopropanol for 60 s. Then exposed graphene was etched away using oxygen plasma (5 Pa and 100 W for 2 s). Electrodes (5 nm Cr and 80 nm Au) were made using another EBL process and electron beam evaporation. The ion–gel dielectric material was prepared by our previous work.^49^

*Characterization of Flexible Graphene Plasmonic Device*: The morphology and size of GNRs were characterized by SEM (Hitachi S‐4800) and AFM (Bruker Dimension Icon). The defects of GNRs were confirmed by Raman spectroscopy (Horiba Jobin Yvon LabRAM HR800). Electrical transport measurements were performed using a semiconductor parameter analyzer (Keithley 4200‐SCS) at room temperature in atmosphere. Infrared transmission measurements were performed by a FTIR microscopy (Thermo Fisher Nicolet iN10) with *T* = 1 − *T_E_*_F_/*T*_CNP_, where *T*_CNP_ and *T_E_*_F_ are the transmission spectra detected at the CNP and *E*_F_ of graphene, respectively. The nanoimaging experiments were performed using a commercial s‐SNOM (Neaspec GmbH), with wavelength‐tunable lasers (900–1000 cm^−1^).

*Simulation Methods*: Electromagnetic simulations were conducted using a Finite Elements Method with periodic boundary conditions. Graphene was modeled as a 2D surface with complex conductivity from Kubo formula which consisted of interband and intraband transitions; the expression is approximated as

(1) $$\sigma =\frac{ie^{2}E_{F}}{\pi \hbar^{2}(\omega +i\tau^{-1})}+\frac{ie^{2}}{4\pi \hbar }ln\left[\frac{2\left|E_{F}\right|-\hbar (\omega +i\tau^{-1})}{2\left|E_{F}\right|+\hbar (\omega +i\tau^{-1})}\right]$$

where the angular frequency is ω=2πυ, e is electron charge, ℏ is the reduced Planck constant. The relaxation time $\tau =\mu E_{F}\mathrm{/e}v_{F}^{2}$, *where v_F_* = *c*/300 is the Fermi velocity and *µ* = 1000 cm^2^ V^−1^ s^−1^ is the carrier mobility of graphene.^58^ *E_F_* is the graphene Fermi energy which is 0.5 eV and the ribbons width *W* of graphene is 60 nm.

## Conflict of Interest

The authors declare no conflict of interest.

## Supporting information

Supplementary

## References

[1] M. B. Lundeberg , Y. Gao , R. Asgari , C. Tan , B. Van Duppen , M. Autore , P. Alonso‐González , A. Woessner , K. Watanabe , T. Taniguchi , Science 2017, eaan2735. 10.1126/science.aan2735 28596312

[2] E. Hwang , S. D. Sarma , Phys. Rev. B 2007, 75, 205418.

[3] T. Low , P. Avouris , ACS Nano 2014, 8, 16. 10.1021/nn406627u 24484181

[4] A. N. Grigorenko , M. Polini , K. S. Novoselov , Nat. Photonics 2012, 6, 749.

[5] H. G. Yan , Z. Q. Li , X. S. Li , W. J. Zhu , P. Avouris , F. N. Xia , Nano Lett. 2012, 12, 3766. 22690695 10.1021/nl3016335

[6] W. Gao , G. Shi , Z. Jin , J. Shu , Q. Zhang , R. Vajtai , P. M. Ajayan , J. Kono , Q. Xu , Nano Lett. 2013, 13, 3698. 23895501 10.1021/nl401591k

[7] L. Ju , B. S. Geng , J. Horng , C. Girit , M. Martin , Z. Hao , H. A. Bechtel , X. G. Liang , A. Zettl , Y. R. Shen , F. Wang , Nat. Nanotechnol. 2011, 6, 630. 21892164 10.1038/nnano.2011.146

[8] S. Kim , M. S. Jang , V. W. Brar , Y. Tolstova , K. W. Mauser , H. A. Atwater , Nat. Commun. 2016, 7, 12323. 27499258 10.1038/ncomms12323 PMC4979088

[9] D. Rodrigo , A. Tittl , O. Limaj , F. J. G. de Abajo , V. Pruneri , H. Altug , Light: Sci. Appl. 2017, 6, e16277. 10.1038/lsa.2016.277 PMC6062234 30167262

[10] F. H. L. Koppens , D. E. Chang , F. J. G. de Abajo , Nano Lett. 2011, 11, 3370. 21766812 10.1021/nl201771h

[11] S. Thongrattanasiri , F. J. G. de Abajo , Phys. Rev. Lett. 2013, 110, 187401. 23683241 10.1103/PhysRevLett.110.187401

[12] D. B. Farmer , D. Rodrigo , T. Low , P. Avouris , Nano Lett. 2015, 15, 2582. 25749426 10.1021/acs.nanolett.5b00148

[13] S. Kim , M. S. Jang , V. W. Brar , K. W. Mauser , L. Kim , H. A. Atwater , Nano Lett. 2018, 18, 971. 29320203 10.1021/acs.nanolett.7b04393

[14] D. N. Basov , M. M. Fogler , F. J. García de Abajo , Science 2016, 354, aag1992. 27738142 10.1126/science.aag1992

[15] T. Low , A. Chaves , J. D. Caldwell , A. Kumar , N. X. Fang , P. Avouris , T. F. Heinz , F. Guinea , L. Martin‐Moreno , F. Koppens , Nat. Mater. 2017, 16, 182. 27893724 10.1038/nmat4792

[16] A. Nikitin , P. Alonso‐González , S. Vélez , S. Mastel , A. Centeno , A. Pesquera , A. Zurutuza , F. Casanova , L. Hueso , F. Koppens , Nat. Photonics 2016, 10, 239.

[17] Y. Li , H. Yan , D. B. Farmer , X. Meng , W. Zhu , R. M. Osgood , T. F. Heinz , P. Avouris , Nano Lett. 2014, 14, 1573. 24528250 10.1021/nl404824w

[18] D. Rodrigo , O. Limaj , D. Janner , D. Etezadi , F. J. García de Abajo , V. Pruneri , H. Altug , Science 2015, 349, 165. 26160941 10.1126/science.aab2051

[19] H. Hu , X. Yang , F. Zhai , D. Hu , R. Liu , K. Liu , Z. Sun , Q. Dai , Nat. Commun. 2016, 7, 12334. 27460765 10.1038/ncomms12334 PMC4974468

[20] D. B. Farmer , P. Avouris , Y. Li , T. F. Heinz , S.‐J. Han , ACS Photonics 2016, 3, 553.

[21] X. Yang , Z. Sun , T. Low , H. Hu , X. Guo , F. J. G. d. Abajo , P. Avouris , Q. Dai , Adv. Mater. 2018, 30, 1704896. 10.1002/adma.201704896 29572965

[22] H. G. Yan , X. S. Li , B. Chandra , G. Tulevski , Y. Q. Wu , M. Freitag , W. J. Zhu , P. Avouris , F. N. Xia , Nat. Nanotechnol. 2012, 7, 330. 22522668 10.1038/nnano.2012.59

[23] H.‐J. Li , L.‐L. Wang , J.‐Q. Liu , Z.‐R. Huang , B. Sun , X. Zhai , Appl. Phys. Lett. 2013, 103, 211104.

[24] M. Polini , Science 2016, 351, 229. 26816365 10.1126/science.aad7995

[25] Z. Sun , A. Martinez , F. Wang , Nat. Photonics 2016, 10, 227.

[26] Z. Fei , A. S. Rodin , G. O. Andreev , W. Bao , A. S. McLeod , M. Wagner , L. M. Zhang , Z. Zhao , M. Thiemens , G. Dominguez , M. M. Fogler , A. H. C. Neto , C. N. Lau , F. Keilmann , D. N. Basov , Nature 2012, 487, 82. 22722866 10.1038/nature11253

[27] J. N. Chen , M. Badioli , P. Alonso‐Gonzalez , S. Thongrattanasiri , F. Huth , J. Osmond , M. Spasenovic , A. Centeno , A. Pesquera , P. Godignon , A. Z. Elorza , N. Camara , F. J. G. de Abajo , R. Hillenbrand , F. H. L. Koppens , Nature 2012, 487, 77. 22722861 10.1038/nature11254

[28] J. Christensen , A. Manjavacas , S. Thongrattanasiri , F. H. Koppens , F. J. García de Abajo , ACS Nano 2011, 6, 431. 22147667 10.1021/nn2037626

[29] P. Alonso‐González , A. Y. Nikitin , F. Golmar , A. Centeno , A. Pesquera , S. Vélez , J. Chen , G. Navickaite , F. Koppens , A. Zurutuza , F. Casanova , L. E. Hueso , R. Hillenbrand , Science 2014, 344, 1369. 24855026 10.1126/science.1253202

[30] W. B. Lu , W. Zhu , H. J. Xu , Z. H. Ni , Z. G. Dong , T. J. Cui , Opt. Express 2013, 21, 10475. 23669904 10.1364/OE.21.010475

[31] X. Zhu , W. Yan , N. A. Mortensen , S. Xiao , Opt. Express 2013, 21, 3486. 23481806 10.1364/OE.21.003486

[32] T.‐H. Xiao , L. Gan , Z.‐Y. Li , Photonics Res. 2015, 3, 300.

[33] S. Szunerits , N. Maalouli , E. Wijaya , J. P. Vilcot , R. Boukherroub , Anal. Bioanal. Chem. 2013, 405, 1435. 23314618 10.1007/s00216-012-6624-0

[34] D. Akinwande , N. Petrone , J. Hone , Nat. Commun. 2014, 5, 5678. 25517105 10.1038/ncomms6678

[35] S. Aksu , M. Huang , A. Artar , A. A. Yanik , S. Selvarasah , M. R. Dokmeci , H. Altug , Adv. Mater. 2011, 23, 4422. 21960478 10.1002/adma.201102430

[36] Q. Wang , K. Xu , Z. Wang , F. Wang , Y. Huang , M. Safdar , X. Zhan , F. Wang , Z. Cheng , J. He , Nano Lett. 2015, 15, 1183. 25603278 10.1021/nl504258m

[37] Z. Xian , Y. Hao , Y. Zhao , S. Song , Colloids Surf., A 2017, 533, 55.

[38] A. Das , S. Pisana , B. Chakraborty , S. Piscanec , S. Saha , U. Waghmare , K. Novoselov , H. Krishnamurthy , A. Geim , A. Ferrari , Nat. Nanotechnol. 2008, 3, 210. 18654505 10.1038/nnano.2008.67

[39] V. W. Brar , M. S. Jang , M. Sherrott , J. J. Lopez , H. A. Atwater , Nano Lett. 2013, 13, 2541. 23621616 10.1021/nl400601c

[40] M. Wu , K. Liu , W. Wang , Y. Sui , X. Bai , E. Wang , Nano Res. 2012, 5, 443.

[41] Z. H. Ni , T. Yu , Y. H. Lu , Y. Y. Wang , Y. P. Feng , Z. X. Shen , ACS Nano 2008, 2, 2301. 19206396 10.1021/nn800459e

[42] C. Sönnichsen , T. Franzl , T. Wilk , G. von Plessen , J. Feldmann , O. Wilson , P. Mulvaney , Phys. Rev. Lett. 2002, 88, 077402. 11863939 10.1103/PhysRevLett.88.077402

[43] N. Liu , M. L. Tang , M. Hentschel , H. Giessen , A. P. Alivisatos , Nat. Mater. 2011, 10, 631. 21572410 10.1038/nmat3029

[44] N. Liu , M. Mesch , T. Weiss , M. Hentschel , H. Giessen , Nano Lett. 2010, 10, 2342. 20560590 10.1021/nl9041033

[45] Z. Fei , M. D. Goldflam , J. S. Wu , S. Dai , M. Wagner , A. S. McLeod , M. K. Liu , K. W. Post , S. Zhu , G. C. A. M. Janssen , M. M. Fogler , D. N. Basov , Nano Lett. 2015, 15, 8271. 26571096 10.1021/acs.nanolett.5b03834

[46] A. Y. Nikitin , F. Guinea , F. García‐Vidal , L. Martín‐Moreno , Phys. Rev. B 2011, 84, 161407.

[47] L. E. Mcneil , M. Grimsditch , J. Phys.: Condens. Matter 1993, 5, 1681.

[48] C. Lee , X. Wei , J. W. Kysar , J. Hone , Science 2008, 321, 385. 18635798 10.1126/science.1157996

[49] H. Hu , F. Zhai , D. Hu , Z. Li , B. Bai , X. Yang , Q. Dai , Nanoscale 2015, 7, 19493. 26530788 10.1039/c5nr05175d

[50] R. Williams , Phys. Rev. 1962, 126, 442.

[51] A. Woessner , M. B. Lundeberg , Y. Gao , A. Principi , P. Alonso‐Gonzalez , M. Carrega , K. Watanabe , T. Taniguchi , G. Vignale , M. Polini , J. Hone , R. Hillenbrand , F. H. Koppens , Nat. Mater. 2015, 14, 421. 25532073 10.1038/nmat4169

[52] X. Yang , F. Zhai , H. Hu , D. Hu , R. Liu , S. Zhang , M. Sun , Z. Sun , J. Chen , Q. Dai , Adv. Mater. 2016, 28, 2931. 26889663 10.1002/adma.201505765

[53] X. Li , S. He , Y. Jin , Phys. Rev. B 2007, 75, 045103.

[54] H. G. Yan , T. Low , W. J. Zhu , Y. Q. Wu , M. Freitag , X. S. Li , F. Guinea , P. Avouris , F. N. Xia , Nat. Photonics 2013, 7, 394.

[55] C. H. Lui , L. Liu , K. F. Mak , G. W. Flynn , T. F. Heinz , Nature 2009, 462, 339. 19924211 10.1038/nature08569

[56] J. Homola , Chem. Rev. 2008, 108, 462. 18229953 10.1021/cr068107d

[57] M. Freitag , T. Low , W. Zhu , H. Yan , F. Xia , P. Avouris , Nat. Commun. 2013, 4, 1951. 23727714 10.1038/ncomms2951

[58] P.‐Y. Chen , A. Alù , ACS Nano 2011, 5, 5855. 21662981 10.1021/nn201622e

