# Supplementary material for: Flexible and Electrically Tunable Plasmons in Graphene–Mica Heterostructures
Source: Adv Sci (Weinh). 2018 Jun 16;5(8):1800175. doi: 10.1002/advs.201800175 (PMC6096988; doi:10.1002/advs.201800175)
Supplement: Supplementary file 1 — Supplementary [file ADVS-5-1800175-s001.pdf]

Copyright WILEY-VCH Verlag GmbH & Co. KGaA, 69469 Weinheim, Germany, 2018.

ADVANCED SCIENCE Open Access

Supporting Information

for *Adv. Sci.,* DOI: 10.1002/advs.201800175

Flexible and Electrically Tunable Plasmons in Graphene–Mica Heterostructures

Hai Hu, Xiangdong Guo, Debo Hu, Zhipei Sun, Xiaoxia Yang, and Qing Dai**

Supporting Information

Flexible and electrically-tunable plasmons in graphene-mica heterostructures

Hai Hu[1,2], Xiangdong Guo[1,2], Debo Hu[1,2], Zhipei Sun[3,4], Xiaoxia Yang[1,2], Qing Dai[1,2]

1 Division of Nanophotonics, CAS Center for Excellence in Nanoscience,

National Center for Nanoscience and Technology, Beijing 100190, P. R. China

2 University of Chinese Academy of Sciences, Beijing 100049, P. R. China,

3 Department of Electronics and Nanoengineering, Aalto University, FI-00076 Aalto, Finland.

4 QTF Centre of Excellence, Department of Applied Physics, Aalto University, FI-00076 Aalto, Finland

Correspondence: daiq@nanoctr.cn; yangxx@nanoctr.cn;

This PDF file includes:

• Figure S1. Schematic diagram of the typical procedure of back-support method for fabricating the flexible graphene plasmonic devices on mica sheets.
• Figure S2. The transfer curve of our flexible device and the dependence of the graphene Fermi levels on gate voltage.
• Figure S3. Numerical calculation of extinction spectra combined with electric charge distribution and field confinement of graphene plasmon on arc surfaces to explore the bending limit of flexible device.
• Figure S4. Photograph of the flexible device and SEM characterization of the GNRs array after 1000 bending cycles.
• Figure S5. Electrical measurement and Raman spectroscopy characterization of the effect on GNRs at different bending cycles.
• Figure S6. Graphene plasmon device based on rigid SiO_2 substrate.
• Figure S7. Fano fitting of plasmon resonance line shape and Lorentz fitting of IR absorption peaks of PEO molecules.
• Figure S8. FTIR spectra of different thickness of mica samples.
• Figure S9. Optical image of monolayer graphene on mica and 285 nm SiO_2 by mechanical exfoliation.
• Figure S10. Experimental extinction spectra of our graphene plasmonic devices with different bending curvature radius at $|E_F|$=0.56 eV.

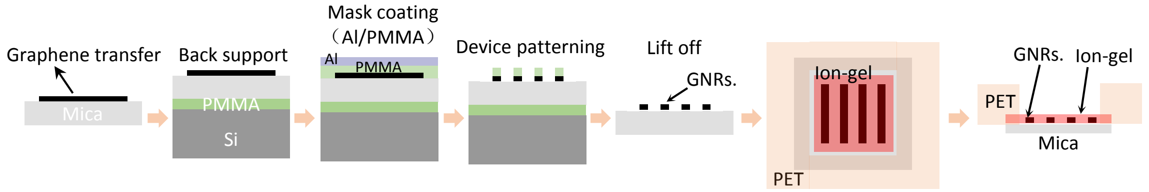

Figure S1. Schematic diagram of the typical procedure of back-support method for fabricating the flexible graphene plasmonic devices on mica sheets.

Graphene is grown on copper films by chemical vapor deposition (CVD) method and then transferred onto the flexible mica substrate following a wet-transfer method. Before the transfer process, a PMMA layer was spin-coated on the upside of graphene/copper foil. Subsequently, the backside graphene was removed using oxygen plasma and the copper foil was selectively etched in 1:1 iron chloride (0.5 mol/L) and hydrochloric acid (0.5 mol/L) solution. The PMMA/graphene film floating on the etchant was cleaned by deionized water several times to rinse the etchant residue and then transferred onto doped Si wafers with 285 nm SiO_2. The chip was left to dry and then the PMMA layer was dissolved by acetone and the whole chip is cleaned by isopropyl alcohol. Next, the wet 950K PMMA film was spin-coated on the upside of Si substrate with 2000 rmp. The graphene/mica sheet was fastened onto a Si substrate spin-coated with wet PMMA film in advance, which acted as supporting plate. The sample was baked at 50 ℃ for 15 min to make the PMMA film was dried completely. Then, another 280 nm 950K PMMA layer is spin coated on the graphene/mica surface as mask and another 15 nm Al film is deposited on it as a conductive layer. Nanoribbon arrays were patterned on PMMA by using 100 keV electron beam lithography (Vistec, EBPG5000). The Al layer was washed away by 238D developing solution. The exposed PMMA was developed in 3:1 isopropanol: methyl isobutyl ketone (MIBK) for 1 min, and then the graphene exposed to air was etched away using oxygen plasma at 5 Pa and 100 W for 2 s. A second electron beam lithography process was carried out to define the

electrode pattern. Devices were then fabricated with contact metal (60 nm-Au/5 nm-Cr) deposition through electron beam evaporation and following a standard metal lift-off technique. The flexible mica device was pasted onto PET film (200 μm in thickness). The PET film is used as fixed mounts to further thin the mica substrate by mechanical exfoliation and to ensure stability of the experimental test. Ion-gel was spin-coated on the device to electrically tune graphene plasmon.

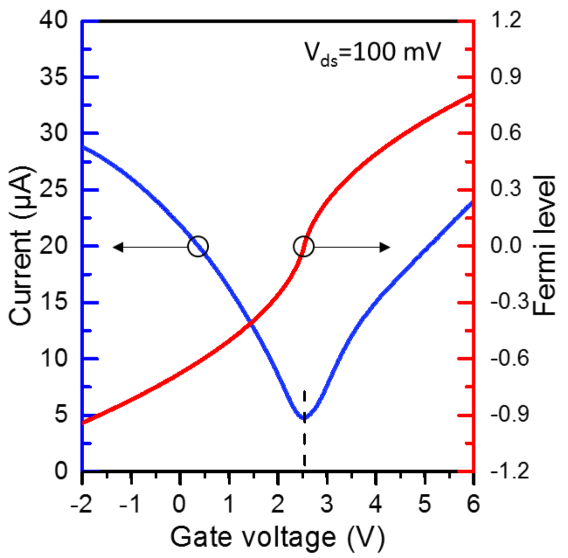

Figure S2. The transfer curve (blue line) of our flexible device. The gate voltage that corresponds to the charge neutral point (CNP, V_{CNP}, marked as dash line) is 2.5 V. The red curve is the dependence of the graphene Fermi levels on gate voltage.

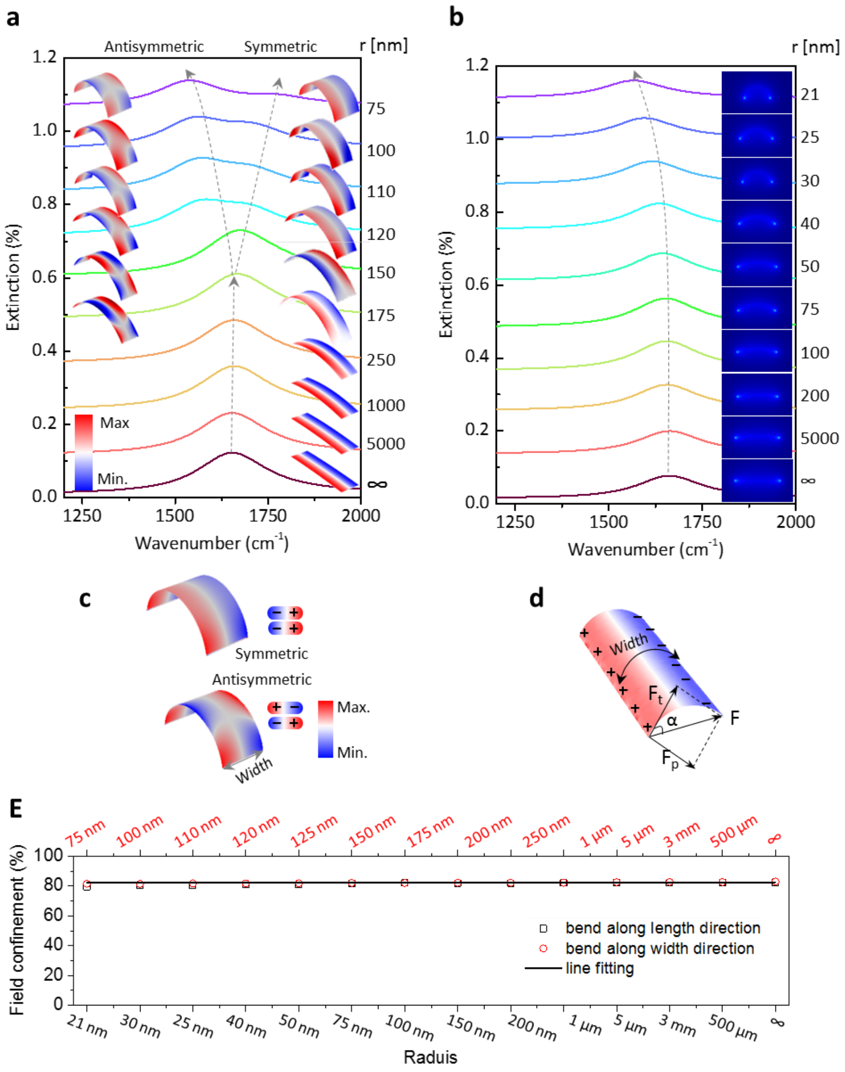

Figure S3. (a), (b) Numerical calculation of extinction spectra of graphene plasmon on arc nanoribbons bending along length and width directions, respectively. The corresponding bending radii are indicated on the right side. **(c), (d)** Electric charge distribution of the plasmonic modes (corresponding to the resonance modes in the far-field extinction spectra) across the arc nanoribbon with a radius of 100 nm along length and width directions, respectively. In (c), the symmetric and antisymmetric modes happen. In (d), F is decomposed into F_t (tangential) and F_P (perpendicular). F_t serves as effective restoring force. **(e)** Percentage of electric field confined in 20 nm considering total near-field intensity extending a distance 20 nm outside nanoribbons.

To understand the reason of high stability, we conduct simulation studies under various bending radii by using a frequency-domain finite-element model. For simplicity, we focus on curved free-standing graphene nanoribbons. Bending along the length and width direction are both considered.

The calculated extinction spectra of the graphene nanoribbons at different bend radii along the length direction and the width direction are plotted in

Figure S3a and S3b, respectively. The variation of resonance frequency, extinction intensity and quality factor of graphene plasmon from simulation are plotted with corresponding experimental results (Figures 2(c-e) in the manuscript). The extinction responses are the same with the flat ribbons when the radius of curvature is larger than 125 nm. This feature is complete accord to the experimental results (Figures 2(b-e) in the manuscript). But, when the radius along the ribbon length direction is smaller than 120 nm, the resonance frequency occurs red shift and two peaks emerge. This is consistent with the near electric field distributions in the curved GNRs. The charge distribution of the plasmon in GNR with large radius shows only one mode, the symmetric one. When the radius of GNR is smaller than 120 nm, two different modes appear, a symmetric mode at lower frequency with the same polarity and antisymmetric mode at higher frequency with opposite polarity (Figure S3c). The antisymmetric mode arises from the coupling effect between spatial overlapped plasmon on the arc surface. As the radius of GNR decreases, this spatial overlap increases and the energy splitting between the symmetric and antisymmetric modes increase. When GNR is curved to the limit condition, graphene is folded as very similar to the stacked two layers and the overlap and electromagnetic coupling of plasmon reach the maximum. As reported in previous study, the near-fields coupling of stacked two graphene layers resulted two resonant peaks above and below the plasmon energy in monolayer layer (Ref. 14). Moreover, the hybridization model can be further evidenced in our simulations by the continuous spectral separation and the shift of the two resonance peaks as the radius progressively approaches 75 nm.

The bending along the width direction has much less effects on the graphene plasmon. Even when the radius is down to 75 nm, the extinction spectra hardly change. When the radius decreases to 40 nm, the resonance frequency of plasmon becomes redshift slightly from 1661 to 1640 cm^{-1} and extinction intensity decreases a little. The simulated charge distribution in real space of the

plasmonic mode is also displayed in Figure S3b. Only the dipolar mode resonant along the curved graphene ribbon is observed. To intuitively understand the redshift with the increased bending, we discuss from the restoring force of the dipole mode. The effective restoring force is along the tangent line for dipole resonance mode on a curved surface, $F_t = F\cos\alpha = F\cos(w/2r)$. Where F is the electric field force along the GNRs on a flat surface, W is the width of ribbons and r is the radius of curvature (Figure S3d). Obviously, F_t is smaller than F and consequently, the oscillation frequency occurs redshift as the radius r continuously decreases. For example, when the radius r decreases from 40 nm to 21 nm, the spectrum is progressive redshift 32 cm^{-1}.

We extract the near-field intensity confinement as a function of the distance d from out-plane of graphene. Figure S3e shows that about 82% of the plasmon energy is confined within a volume extending a distance 20 nm outside nanoribbon, which is almost irrelevant to bending deformation of GNRs. The ultra-strong field confinement keeps the spatial overlap of plasmon in curved graphene low, which results high stability of the plasmonic devices with radius above 125 nm, as discussed above.

We also calculate that the limit radius of curvature for flexible graphene plasmon with stability. The curvature-induced radiative energy loss will affect the plasmons on curved graphene surface. Hence, there exists a critical curvature radius r* that the electromagnetic fields can no longer be confined. The critical curvature radius can be calculated by:

$$r^* = \frac{k_0}{\mathrm{Re}(\sqrt{\beta^2 - k_0^{\,2}}) \cdot (\beta - k_0)}$$

where k_0 is free space wave vector. β is a parameter closely related to the conflnement (Ref: Physical Review A 75.6 (2007): 063816.; Ref: PIERS Proceedings. 2014.; Ref: Optics express 21.9 (2013): 10475-10482.). The calculated r* for graphene is $r^* = 0.02\lambda_p$.

In our experiments, plasmon with multiple reflections between the two edges of GNRs leads to constructive interference, similar to Fabry-Perot resonances, which can be described as,

$$2\,\mathrm{Re}\,(k_p)\,W + \phi = 2m\pi$$

Where k_p is the wavevector of graphene plasmon and ϕ ($0.6\pi<\phi<0.7\pi$) is the phase shift of the plasmon upon reflection. For the 65 nm width of GRNs and plasmon resonant energies at 1300 cm^{-1}, $\lambda_p=2\pi/\mathrm{Re}\,(k_p)=150$ nm. Then the smallest critical curvature radius r*=3 nm.

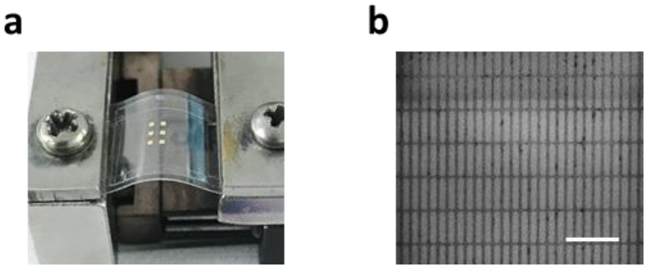

Figure S4. (a) Photograph of the flexible device after 1000 bending cycles. **(b)** SEM characterization of the GNRs array after 1000 bend cycles.

After repeated bending of 1000 cycles in 75 min, the morphology of GNRs is in good shape without measurable performance degradation

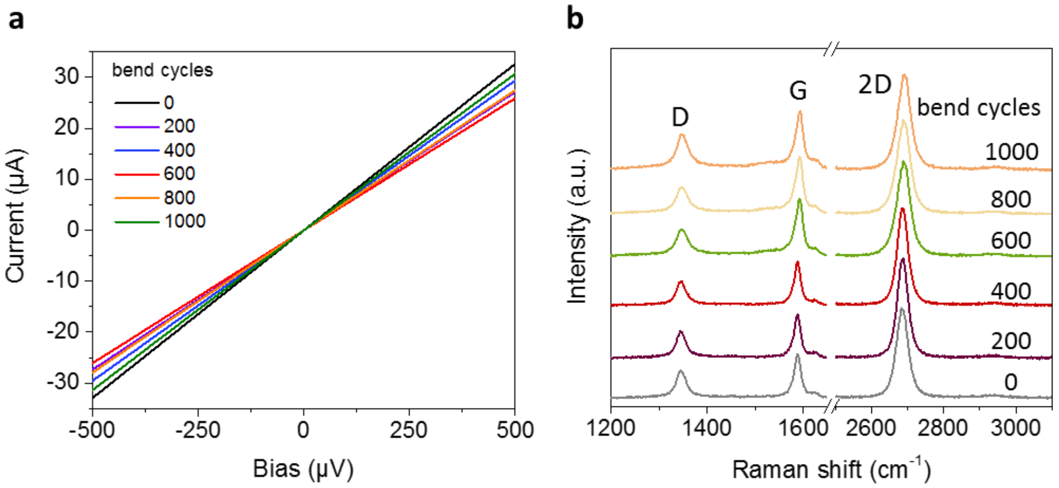

Figure S5. (a), (b) electrical measurement and Raman spectroscopy characterization of the effect on GNRs at different bending cycles.

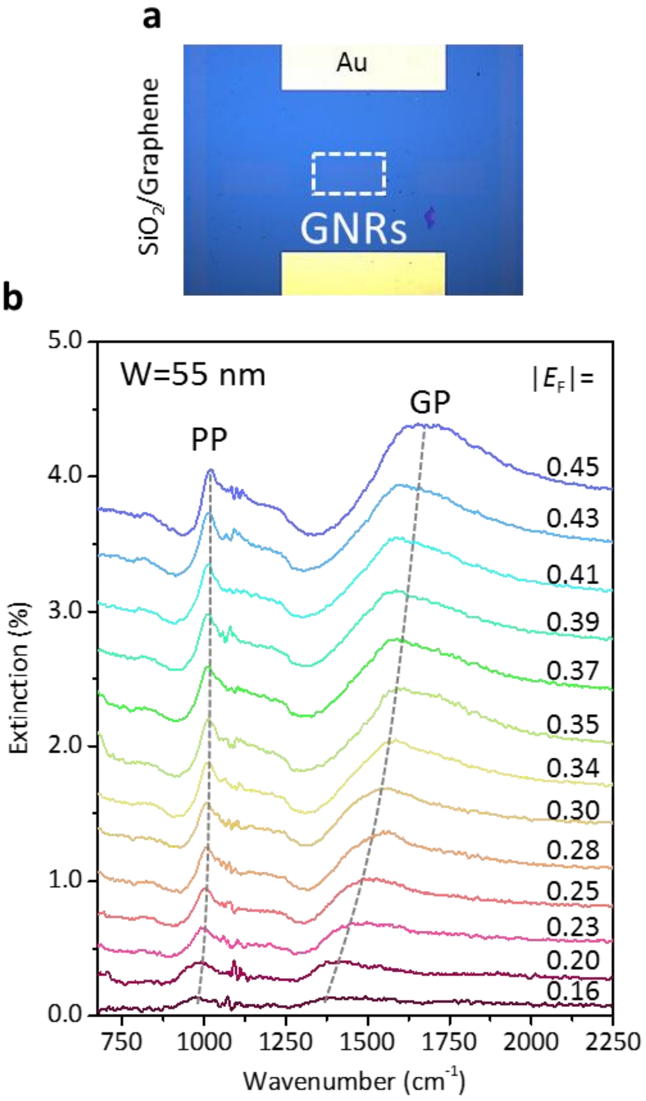

Figure S6. (a) Optical micrograph of a typical graphene plasmon device on SiO_2/Si substrate. **(b)** Extinction spectra of the graphene nanoribbon array (55 nm) at various values of E_F controlled by the back gate via SiO_2 dielectric layer, respectively.

There are two distinct resonance peaks in the extinction spectra which are ascribed to the coupling of graphene plasmons to two surface optical (SO) phonons of SiO_2 at 806 cm^{-1} and 1168 cm^{-1}, respectively. These plasmon-phonon hybridization modes are from destructive interference when the graphene plasmons and vibrational modes interact with an opposite phase relationship.

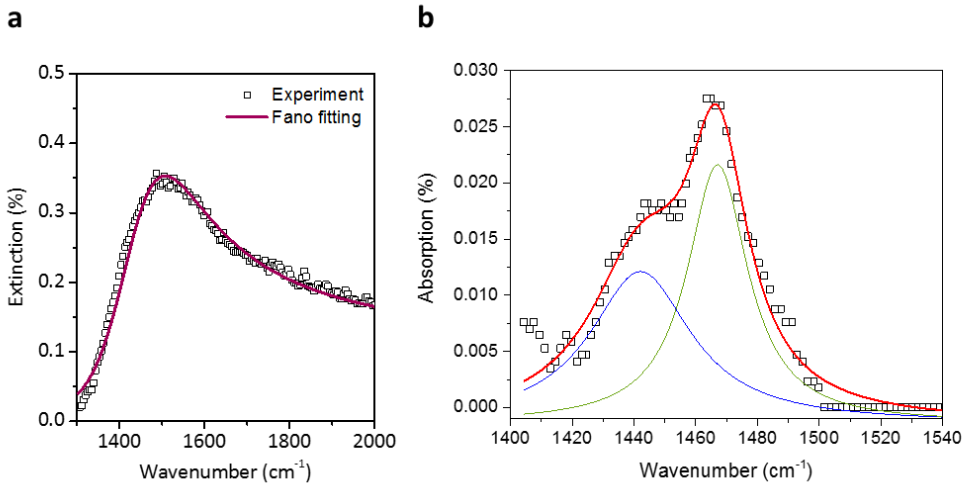

Figure S7. (a) Resonance line shape and fitting. Measured extinction spectra of a $W = 55$ nm ribbon array on mica substrate. The data can be fitted well by a Fano model. **(b)** Extracted absorption feature of PEO vibrational modes from experimental extinction spectra of graphene plasmon. The Lorentz line shapes (the thin solid blue and green curves) are used to fit the IR absorption peaks of PEO molecules. The red curve indicates the superposition of the fitted peak.

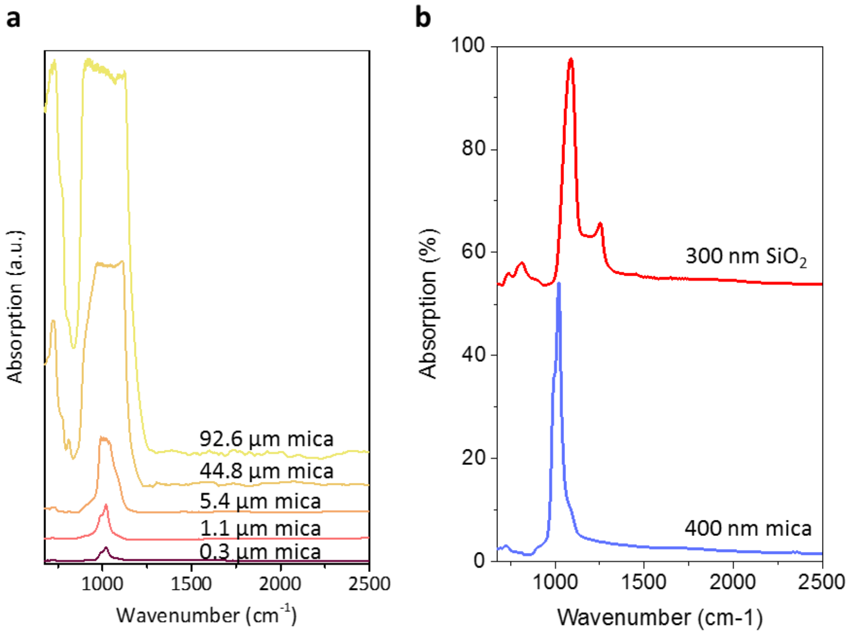

Figure S8. (a) FTIR spectra of different thickness of mica samples. **(b)** A comparison of the IR absorption spectra between 300 nm thick SiO2 and 308 nm mica.

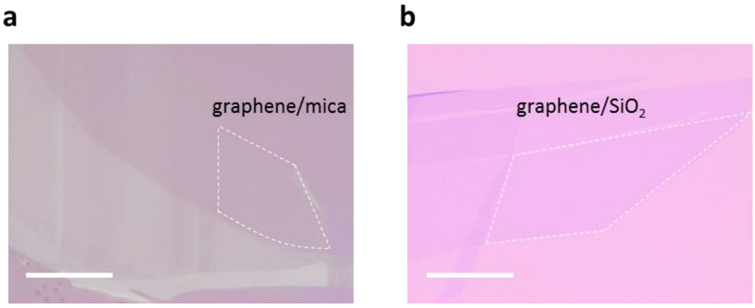

Figure S9. (a), (b) Optical image of monolayer graphene on mica and 285 nm SiO_2 by mechanical exfoliation, respectively. The scale bar is 15 μm. For better contrast under microscope, the graphene/mica sheet was put on 285 nm SiO_2/500 μm Si substrate.

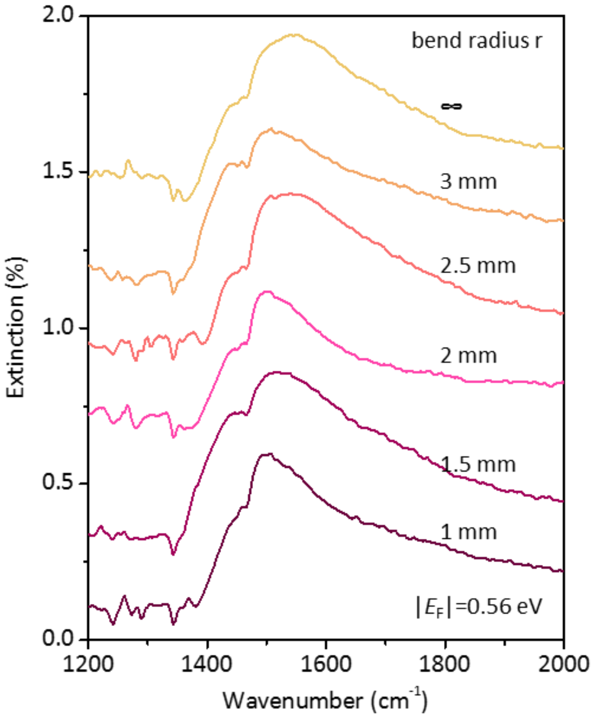

Figure S10. Experimental extinction spectra of our graphene plasmonic devices with different bending curvature radius at $|E_F|$=0.56 eV.
